# DNA mismatch repair mediated by Mlh1–Pms1 endonuclease-catalyzed mispair excision

**DOI:** 10.1073/pnas.2528670122

**Published:** 2025-12-24

**Authors:** Tatiana Palacio, Felipe A. Calil, Nikki Bowen, Jack D. Griffith, Christopher D. Putnam, Richard D. Kolodner

**Affiliations:** ^a^Department of Cellular and Molecular Medicine, University of California San Diego School of Medicine, La Jolla, CA 92093-0660; ^b^Program in Virology, Lineberger Cancer Center, University of North Carolina at Chapel Hill, Chapel Hill, NC 27514; ^c^Department of Microbiology and Immunology, University of North Carolina at Chapel Hill, Chapel Hill, NC 27514; ^d^Department of Pediatrics, University of California San Diego School of Medicine, La Jolla, CA 92093-0660; ^e^Structural and Functional Genomics Program, Moores–University of California San Diego Cancer Center, University of California San Diego School of Medicine, La Jolla, CA 92093-0660; ^f^Institute of Genomic Medicine, University of California San Diego School of Medicine, La Jolla, CA 92093-0660

**Keywords:** DNA replication, mutation suppression, Msh2-Msh6, Msh2-Msh3, exonuclease 1

## Abstract

Eukaryotic DNA mismatch repair (MMR) involves several excision pathways, including those mediated by exonuclease 1 (Exo1) and by the flap endonuclease Rad27 (human FEN1) coupled with DNA polymerase δ. Simultaneous inactivation of both excision mechanisms causes an MMR defect that is at most 5 to 13% of that caused by complete inactivation of MMR. Here, we reconstituted nicked-strand-specific MMR with the *Saccharomyces cerevisiae* proteins Msh2-Msh6 or Msh2-Msh3, DNA polymerase ε, RFC, PCNA, RPA, and Mlh1-Pms1 (human Mlh1-Pms2) under conditions lacking Exo1, Rad27, or strand-displacement synthesis by DNA polymerase δ. These reactions required the Mlh1-Pms1 endonuclease activity, its activation by RFC and PCNA, and its recruitment by Msh2-Msh6 or Msh2-Msh3. MMR was mediated by nicked-strand-specific excision by Mlh1-Pms1 through formation of single-strand DNA gaps having a broad range of sizes. This reaction is consistent with genetic data demonstrating redundancy between the Exo1, Rad27, and Mlh1-Pms1 excision pathways in MMR.

## Significance

DNA mismatch repair (MMR) prevents mutations due to DNA replication errors and suppresses the development of cancers. Previously identified mispair excision pathways dependent on either Exonuclease 1 or Rad27 only appear to account for a small fraction of MMR. Here, we identified and reconstituted an Mlh1-Pms1 endonuclease-mediated excision reaction that along with the Exonuclease 1 and Rad27 excision pathways appears to account for most or all of MMR. These results identify an unappreciated role for the Mlh1-Pms1 endonuclease in directly catalyzing mispair excision that provides a comprehensive view of the range of excision pathways that function in MMR and also provides an explanation for the universal requirement for Mlh1-Pms1 in MMR.

Eukaryotic DNA mismatch repair (MMR) plays different roles in cells including: 1) repair of mispaired nucleotides resulting from DNA replication errors and some types of chemical damage to DNA that can lead to mutations; 2) processing of mispaired bases formed in heteroduplex recombination intermediates that can lead to gene conversion; and 3) suppression of recombination between divergent DNA sequences that can lead to genome rearrangements (1–7). MMR also plays a role in the DNA damage response (8). Because MMR prevents mutations due to errors in DNA replication, MMR suppresses the development of cancer, and MMR defects underlie both inherited cancer susceptibility and spontaneous cancers (9–11).

Our understanding of eukaryotic MMR mechanisms comes from a combination of genetic studies, biochemical characterization of individual MMR proteins and biochemical reconstitution of MMR reactions (1–4). One of the essential core MMR components are the MutS-related heterodimeric Msh2-Msh6 and Msh2-Msh3 mispair recognition complexes that have different mispair binding specificity, recruit other MMR proteins and are coupled to DNA replication (12–18). The other essential core MMR component is the MutL-related heterodimeric Mlh1-Pms1 complex (called Mlh1-Pms2 in humans), which is an endonuclease that also recruits other MMR proteins (19–26). Two other MutL related complexes, the Mlh1-Mlh3 endonuclease and the Mlh1-Mlh2 complex, which lacks an endonuclease active site, potentially play minor roles in MMR (27–29). Other proteins which act in MMR include Exo1, Rad27, PCNA, RFC, RPA, DNA polymerase δ (DNA Polδ) and DNA polymerase ε (DNA Polε) (30–35), as well as potentially the chromatin remodeling Fun30 protein and RSC complex (19, 36).

Biochemical reconstitution studies have primarily focused on repair of circular substrates containing a mispair and a single-stranded nick 5′ to the mispair (13, 30–32, 34, 35). In these reactions, Msh2-Msh6 or Msh2-Msh3 appear to recruit Exo1 that then excises the nicked strand starting at the 5′ nick until the mispair is excised; excision is thought to be modulated by RPA (34, 37). The gap is filled in by either DNA Polδ in combination with PCNA and RFC or DNA Polε, resulting in a nicked product that can be sealed with DNA ligase (30, 33). When the circular substrate contains a single-stranded nick 3′ to the mispair, the Mlh1-Pms1 endonuclease is recruited by Msh2-Msh6 or Msh2-Msh3 and makes a nick or nicks 5′ to the mispair only in the already nicked strand, allowing MMR to proceed by the Exo1-dependent MMR reaction (21, 23, 38). The mechanism by which Mlh1-Pms1 only nicks the already nicked strand is not yet clear, although it could involve PCNA or product recognition by the Mlh1-Pms1 endonuclease (22, 38–40). While these Exo1-dependent excision reactions act in MMR, they cannot fully account for MMR in vivo: 1) deletion of the *EXO1* gene causes only a small MMR defect that is less than 1% of the defect caused by deleting *MSH2* or *MLH1* (15, 33, 41); and 2) since replicating DNA already contains leading and lagging strand nicks, providing nicks for Exo1 excision may not be the essential role for the Mlh1-Pms1 endonuclease in MMR (42).

To better understand Exo1-independent MMR, we have previously conducted genetic screens and biochemical experiments to identify Exo1-independent MMR pathways. We initially defined two MMR pathways, an Exo1-dependent pathway and an Exo1-independent pathway that is more sensitive to defects in Mlh1-Pms1 activity (26, 38, 41, 43). Analysis of these pathways provided insights into recruitment of Exo1 to MMR and the activation of the Mlh1-Pms1 endonuclease suggesting the Exo1-independent pathway requires higher levels of Mlh1-Pms1 activity (19, 38, 43). A second Exo1-independent pathway, Rad27-dependent MMR, was not initially identified by these studies as *exo1Δ* and *rad27Δ* mutations are synthetically lethal (44); however, the development of MMR defective *exo1* mutations that are not synthetically lethal with *rad27Δ* mutations (19) led to genetic evidence for a Rad27-dependent MMR pathway (33). This result allowed for reconstitution of an MMR reaction in which Rad27, Msh2-Msh6, or Msh2-Msh3, PCNA, RFC, RPA, and DNA Polδ catalyze repair of circular mispair-containing substrates that contain a single-stranded nick 5′ to the mispair (33). Genetic analysis of mutator phenotypes resulting from loss of both Exo1 and Rad27 suggest that together the Exo1- and Rad27-mediated MMR pathways can account for at most 5 to 13% of MMR in otherwise wild-type cells, while all three MMR pathways appear to account for all or most of MMR (33). However, previous studies have not provided insight into the mechanism for the MMR pathway that is independent of Exo1 and Rad27 but dependent on high levels of the Mlh1-Pms1 endonuclease (19, 38, 43). We therefore investigated the ability of Mlh1-Pms1 to mediate MMR in vitro in the absence of Exo1 and Rad27 and have reconstituted MMR reactions in which the Mlh1-Pms1 endonuclease directly generates single-stranded DNA gaps that excise the mispair from the DNA strand marked by a preexisting nick. These Mlh1-Pms1 generated gaps can then be filled by the action of either DNA Polδ or DNA Polε.

## Results

### Mlh1-Pms1 Can Mediate an MMR Reaction Independent of Excision by Exo1 and Rad27.

**Fig. 1.**
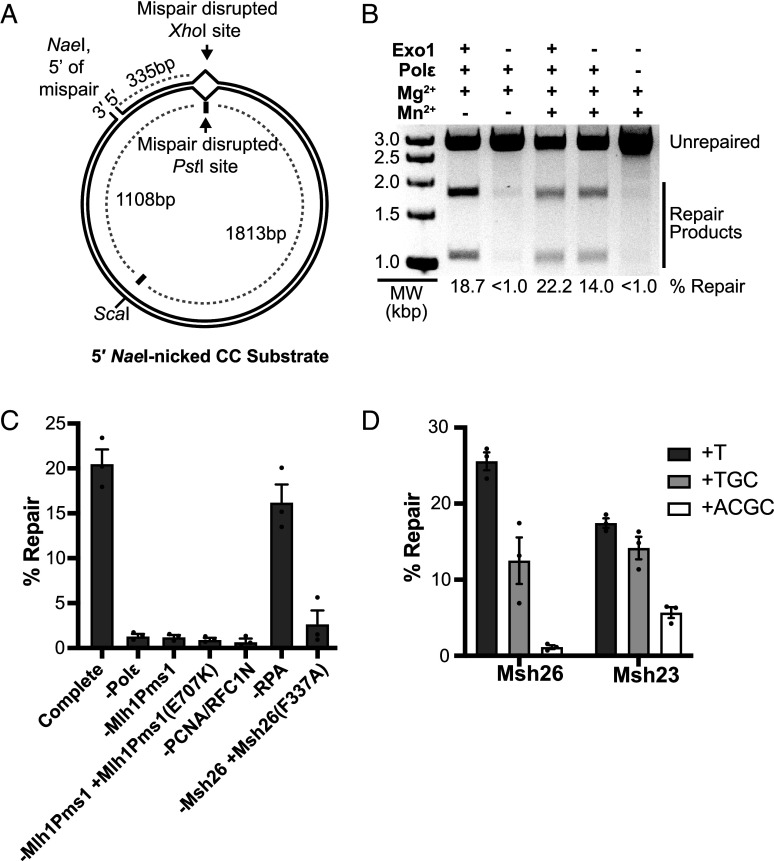
5′ nick-directed Mlh1-Pms1-dependent MMR in a reconstituted system. (*A*) The 5′ nicked CC plasmid substrate contains a CC mispair (*SI Appendix*, Fig. S1) that disrupts a *Pst*I site in the continuous strand and an *Xho*I site in the nicked strand, a 5′ nick at the *Nae*I site and a mispair-distal *Sca*I site. (*B*) Repair reactions containing the 5′ nicked CC mispair substrate, Msh2-Msh6, Mlh1-Pms1, PCNA, RFC-Δ1N, RPA, and Mg^2+^ in which DNA Polε, Exo1, and Mn^2+^ were added as indicated were incubated at 30 °C for 3 h. Repair was detected by the observation of 1.8 and 1.1 kbp fragments by agarose gel electrophoresis after digestion of reaction products with *Sca*I and *Pst*I. MW, molecular weight markers. (*C*) Quantification of Mlh1-Pms1-dependent MMR repair in reactions containing Msh2-Msh6, Mlh1-Pms1, PCNA, RFC-Δ1N, RPA, DNA Polε, Mg^2+^, and Mn^2+^ where components were omitted or substituted as indicated; the bars are the average values, the whiskers indicate the SEM and the dots indicate the values from (n ≥ 3) independent reactions. (*D*) Repair of plasmid substrates containing a +T, +TGC, or +ACGC mispair (*SI Appendix*, Fig. S1) and a 5′ nick at the *Nae*I site in reactions containing Mlh1-Pms1, PCNA, RFC-Δ1N, RPA, DNA Polε, Mg^2+^, and Mn^2+^ and either Msh2-Msh6 or Msh2-Msh3. The reactions were performed and analyzed as described in panels *B* and *C*.

We initially investigated reconstitution of Exo1- and Rad27-independent MMR using a plasmid substrate with a nick at the *Nae*I site that is 5′ to a CC mispair. The CC mispair disrupts a *Pst*I restriction site in the continuous strand, and repair using the continuous strand as template restores this site (Fig. 1*A* and *SI Appendix*, Fig. S1). Consistent with previous results (31, 32, 38), this substrate can be repaired in an Exo1-dependent reaction that also contains Msh2-Msh6, Mlh1-Pms1, PCNA, RFC-Δ1N, RPA, DNA Polε, and Mg^2+^ (Fig. 1*B*). DNA Polε was used in these experiments because it does not catalyze strand displacement synthesis, whereas DNA Polδ does catalyzes strand displacement synthesis, which can generate a biochemical artifact that mimics MMR (45–47). Repair of the substrate was Exo1-dependent in the presence of Mg^2+^. In contrast, the Exo1 dependence was eliminated by adding Mn^2+^, which activates the Mlh1-Pms1 endonuclease (21–23, 38) as can Zn^2+^ (38) (also see legend to Fig. 5*F*) at concentrations seen in cells (48, 49).

To further characterize the Exo1- and Rad27-independent repair reaction, we investigated the requirement of each protein in reactions containing Mg^2+^ and Mn^2+^ (Fig. 1*C*). Repair depended upon mispair recognition, as substitution of Msh2-Msh6 with the mispair recognition-defective Msh2-Msh6(F337A) mutant complex (32, 33, 38, 50) caused a profound repair defect. Repair also required the Mlh1-Pms1 endonuclease activity, as repair was eliminated by omitting Mlh1-Pms1, replacing Mlh1-Pms1 with the endonuclease-defective Mlh1-Pms1(E707K) (21, 23, 26, 38), or omitting PCNA and RFC-Δ1N, which are required to load PCNA for activation of the Mlh1-Pms1 endonuclease (21–23, 43, 51). Repair was also not observed when Mlh1-Pms1 was replaced with Mlh1(R401A, D403A)-Pms1 (*SI Appendix*, Fig. S2), which lacks a functional conserved motif within the unstructured Mlh1 linker that promotes Mlh1-Pms1 endonuclease activity (52). Omitting DNA Polε, which resynthesizes the nicked substrate strand to recreate the *Pst*I site, also eliminated repair. Omitting RPA only modestly reduced the levels of repair, similar to MMR reactions containing Exo1 and DNA Polε (31).

Repair of +T, +TGC, and +ACGC mispairs was also tested (Fig. 1*D* and *SI Appendix*, Fig. S1). When the reactions contained Msh2-Msh6, we observed efficient repair of the +T and +TGC mispairs but little or no repair of the +ACGC mispair. In contrast, when Msh2-Msh3 was substituted for Msh2-Msh6, repair of the +T mispair was somewhat reduced, whereas repair of the +ACGC mispair was increased. These results are consistent with the mispair binding specificity of Msh2-Msh6 and Msh2-Msh3 (12–14, 16). Taken together, these data indicate repair of the 5′ nick-containing substrate under these conditions does not require the previously characterized excision enzymes Exo1 and Rad27 but does depend on 1) mispair recognition by Msh2-Msh6 or Msh2-Msh3, 2) the Mlh1-Pms1 endonuclease activity, and 3) DNA synthesis by DNA Polε.

### Mlh1-Pms1 Mediated MMR Reactions Can Repair Substrates with 3′ Nicks.

**Fig. 2.**
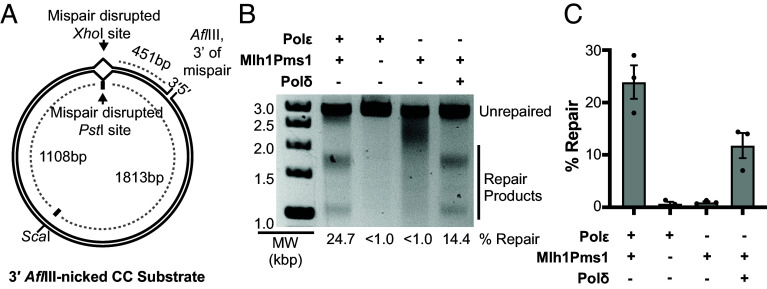
DNA Polε and DNA Polδ can function in 3′ nick-directed Mlh1-Pms1-dependent MMR in a reconstituted system. (*A*) The 3′ nicked CC plasmid substrate contains a CC mispair (*SI Appendix*, Fig. S1) that disrupts a *Pst*I site in the continuous strand and an *Xho*I site in the nicked strand, a 3′ nick at the *Afl*III site, and a mispair-distal *Sca*I site. (*B*) Repair reactions containing the 3′ nicked CC mispair substrate, Msh2-Msh6, Mlh1-Pms1, PCNA, RFC-Δ1N, RPA, DNA Polε, Mg^2+^, and Mn^2+^ in which DNA Polε and Mlh1-Pms1 were omitted and DNA Polδ was added as indicated were incubated at 30 °C for 3 hr. Repair products were detected and quantified as described in the legend to Fig. 1. MW, molecular weight markers. (*C*) The average value for the percent of substrate repaired was quantified as described in the legend to Fig. 1.

The Exo1 exonuclease excises one strand of a double-stranded DNA with 5′-to-3′ polarity (15, 53) and readily excises mispairs from substrates with nicks 5′ to the mispair in reactions containing appropriate additional proteins (30–32, 34, 35). Exo1 mediates the repair of substrates with nicks 3′ to the mispair under two conditions: 1) at high Exo1 levels, long excision tracts starting at the 3′ nick can ultimately reach the mispair due to the circular nature of the substrate (32); and 2) at low Exo1 levels, Exo1-mediated repair depends on strand-specific nicks 5′ of the mispair generated by Mlh1-Pms1 (21, 23, 38). We therefore investigated whether the Exo1- and Rad27-independent reaction could repair a CC mispair-containing substrate with a nick that was 3′ to the mispair (Fig. 2*A*). A reaction containing Msh2-Msh6, PCNA, RFC-Δ1N, and RPA, Mlh1-Pms1, DNA Polε, Mg^2+^, and Mn^2+^ catalyzed significant repair of this substrate, whereas omitting DNA Polε or Mlh1-Pms1 eliminated repair (Fig. 2*B*). Substituting DNA Polε with low levels of DNA Polδ also supported repair in the absence of Exo1 and Rad27 and in the presence of Mlh1-Pms1, Mg^2+^, and Mn^2+^, but this repair was less efficient than the DNA Polε-containing reactions (Fig. 2*B*). Previous studies showed that low levels of DNA Polδ like those used here do not catalyze sufficient strand displacement synthesis to reach the mispair site with substrates containing a 3′ nick (32). Thus, the Exo1- and Rad27-independent, Mlh1-Pms1-dependent reaction can also repair 3′ nicked CC mispair-containing substrates using either DNA Polδ or DNA Polε.

### Mlh1-Pms1 Mediated MMR Reactions Use the Continuous Strand As a Template.

**Fig. 3.**
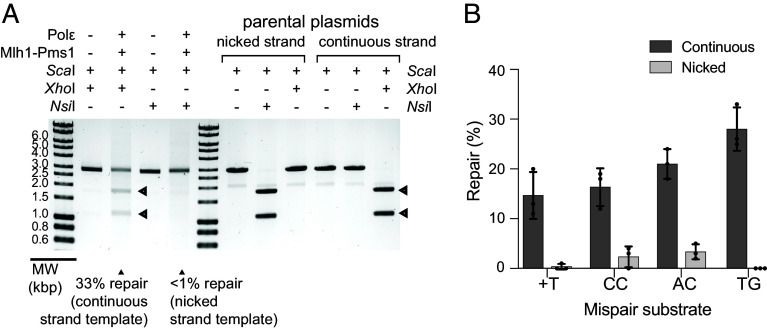
Mlh1-Pms1-dependent MMR exclusively excises and repairs the nicked substrate strand mispaired base. (*A*) (*Left*) Repair reactions containing the 5′ nicked TG mispair substrate and Msh2-Msh6, Mlh1-Pms1, PCNA, RFC-Δ1N, RPA, DNA Polε, Mg^2+^, and Mn^2+^ in which DNA Polε and Mlh1-Pms1 were present or absent were incubated at 30 °C for 2 h. Excision and resynthesis of the nicked strand was detected by digestion with *Sca*I and *Xho*I and excision and resynthesis of the continuous strand was detected by digestion with *Sca*I and *Nsi*I followed by analysis by agarose gel electrophoresis. MW, molecular weight markers. (*Right*) Digestion of control parental plasmids that provide the nicked strand containing the T of the mispair and the *Nsi*I site (pRDK506) and continuous strand containing the G of the mispair and the *Xho*I site (pRDK505) to the 5′ nicked TG mispair substrate were analyzed by digestion with *Sca*I and either *Nsi*I or *Xho*I as indicated. (*B*) Repair reactions containing 5′ nicked substrates containing +T, TG, CC, and AC (*SI Appendix*, Fig. S1) were performed and analyzed as described in panel A. Excision and repair of the nicked strand was detected by digestion with *Sca*I and *Pst*I (+T, CC mispairs), *Xho*I (TG mispair), or *Nsi*I (AC mispair) and excision and repair of the continuous strand was detected by digestion with *Sca*I and *Nsp*I (+T mispair), *Nsi*I (TG mispair), or *Xho*I (CC and AC mispairs). The average value for the percent of the nicked and continuous substrate strands repaired was quantified as described in the legend to Fig. 1.

To ensure that the Mlh1-Pms1 mediated MMR reactions are bona fide MMR reactions that only excise the nicked strand (2, 4, 42), we tested the directionality of repair of a variety of mispaired substrates in MMR reactions containing Msh2-Msh6, Mlh1-Pms1, PCNA, RFC-Δ1N, RPA, DNA Polε, Mg^2+^, and Mn^2+^. We first tested a TG mispair-containing substrate where the mispair disrupts a *Xho*I site on the continuous strand and an *Nsi*I site on the nicked strand (Fig. 3*A* and *SI Appendix*, Fig. S1). The digestion pattern of repair products matched that of the continuous strand donor plasmid but not the nicked strand donor plasmid, indicating that repair specifically excises the nicked strand and uses the continuous strand as a template. We subsequently tested the directionality of repair of +T, CC, and AC mispair-containing substrates (*SI Appendix*, Fig. S1) and similarly found that the digestion pattern of the repaired products only matched that of the continuous strand donor plasmid (Fig. 3*B*). These data show that Mlh1-Pms1-dependent MMR has an absolute polarity with mispair excision occurring on the nicked substrate strand.

### Strand-Specific Nicks Generated by Mlh1-Pms1 Can Rescue PstI Cleavage At Mispairs.

In some control experiments, we found that a small fraction of the +T mispair-containing substrate incubated in reactions lacking a DNA polymerase could be cleaved by *Pst*I (*SI Appendix*, Fig. S3*A*). We hypothesized that *Pst*I cleavage of the mispaired substrate could be promoted by mispair-adjacent nicks generated by Mlh1-Pms1. We therefore generated a series of double-stranded DNA substrates by annealing oligonucleotides whose sequences were derived from our mispair-containing plasmids (*SI Appendix*, Fig. S3*B* and Tables S1 and S2). *Pst*I could not cleave the intact +T heteroduplex substrate but could cleave most substrates with nicks on the “top strand” (+T-containing) within 5 nucleotides of the mispair and some substrates with top strand gaps (*SI Appendix*, Fig. S3*C*). In contrast, a substrate with a nick on the “bottom strand” (insertion-lacking) did not rescue *Pst*I cleavage (*SI Appendix*, Fig. S3*C*). A small number of substrates with a CC mispair and top strand nicks were also tested, and these were cleaved by *Pst*I, although much less efficiently than for the nicked +T mispair substrates (*SI Appendix*, Fig. S3*C*). This suggests that in the reconstituted MMR reactions, Mlh1-Pms1 can cleave the top strand at or near the mispair and *Pst*I cleaves the bottom strand in a reaction that could be dependent or independent of top strand cleavage. To further investigate *Pst*I cleavage, supercoiled plasmids containing a +T mispair or a CC mispair at the *Pst*I site were digested with *Pst*I. The supercoiled +T mispair-containing plasmid was efficiently converted to nicked circles by *Pst*I, whereas the CC mispair-containing plasmid was not (*SI Appendix*, Fig. S3*D*). Together, these results indicate that 1) *Pst*I has half-site reactivity that nicks the bottom strand independently of Mlh1-Pms1 with greater activity on the +T substrate than the CC substrate and that 2) Mlh1-Pms1 facilitates double-stranded cleavage by *Pst*I through generation of mispair-adjacent nicks in the top strand. Together, these results suggest that nicks at or near mispairs catalyzed by Mlh1-Pms1 can contribute to MMR.

### Mlh1-Pms1 Catalyzes the Formation of Single-Strand Gaps.

**Fig. 4.**
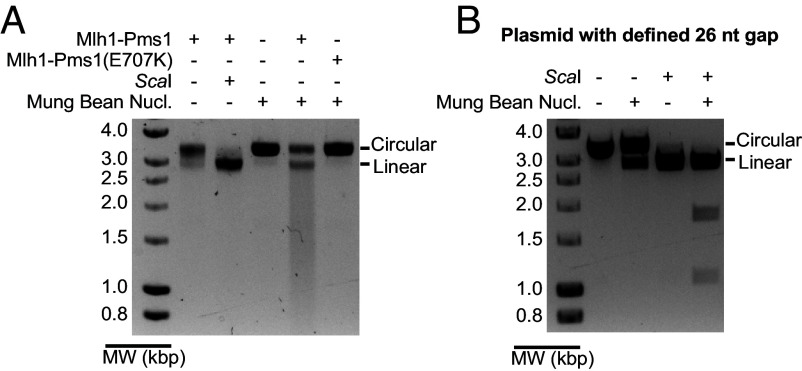
Mlh1-Pms1 generates gaps on a plasmid substrate containing a +T mispair and a 5′ nick at the *Nae*I site. (*A*) Reactions containing the 5′ nicked +T mispair substrate, Msh2-Msh6, Mlh1-Pms1, PCNA, RFC-Δ1N, RPA, Mg^2+^, and Mn^2+^ in which Mlh1-Pms1 was omitted and the Mlh1-Pms1(E707K) active site mutant complex was added as indicated were incubated at 30 °C for 3 h. The DNA products were then digested with *Sca*I or Mung Bean Nuclease as indicated followed by analysis by agarose gel electrophoresis. MW, molecular weight markers. (*B*) *Sca*I and Mung Bean Nuclease digestion of a DNA substrate constructed to have a defined 26 nucleotide gap; the gap spans the mispair-containing region of the mispaired substrates. nt, nucleotides.

To understand how mispair excision occurs without excision enzymes like Exo1 and Rad27 or a strand-displacing DNA polymerase like DNA Polδ, we characterized the DNA products formed by Mlh1-Pms1 in excision reactions lacking a DNA polymerase (Fig. 4*A*). The excision products were circular and could be linearized by *Sca*I digestion. Treatment of these excision products with Mung Bean Nuclease, which cleaves single-stranded gaps but not single-stranded nicks (54), cleaved a portion of the excision products to unit-length linear DNA and a mixture of smaller, more rapidly migrating fragments (Fig. 4*A*; also see *SI Appendix*, Fig. S3*A*). The ability of Mung Bean Nuclease to cleave the DNA products depended upon the Mlh1-Pms1 endonuclease, as products were resistant to digestion when Mlh1-Pms1 was omitted from the reactions or was replaced with the Mlh1-Pms1(E707K) endonuclease active site mutant (21, 23, 26, 38). As a control, we generated a circular DNA substrate containing a defined 26 nucleotide single-stranded gap. A portion of this control substrate was linearized by Mung Bean Nuclease, and the fragment sizes from a Mung Bean Nuclease and *Sca*I double digest (1.1 and 1.8 kb) were consistent with the positions of the gap and the *Sca*I site (Fig. 4*B*). Remarkably, Mung Bean Nuclease digestion of the control substrate did not generate the more rapidly migrating fragments observed after Mung Bean Nuclease digestion of the DNA products formed by Mlh1-Pms1 digestion of the mispair-containing substrate in reactions lacking a DNA polymerase (Fig. 4*B*). These results suggest that the Mlh1-Pms1 endonuclease activity generates multiple single-strand gaps in the substrate DNA and/or gaps of varying sizes.

**Fig. 5.**
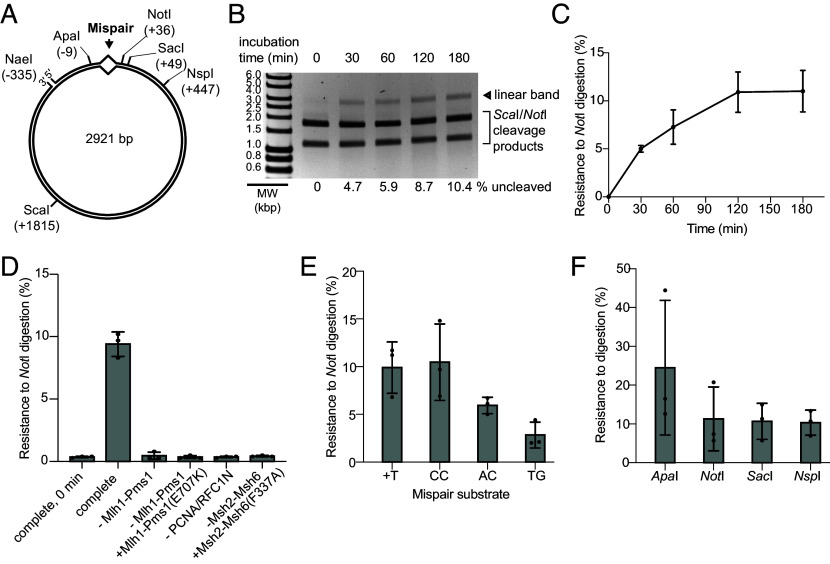
Products of Mlh1-Pms1 excision reactions are resistant to cleavage at mispair-adjacent restriction sites. (*A*) Map of the plasmid-based substrates showing the positions of the mispair, the *Nae*I site 5′ nick, the mispair-distal *Sca*I site and the mispair-adjacent *Apa*I, *Not*I, *Sac*I, and *Nsp*I sites. The sequence of the mispair-containing linker regions for all plasmid substrates are shown in *SI Appendix*, Fig. S1. (*B*) Reactions containing the 5′ nicked +T mispair substrate, Msh2-Msh6, Mlh1-Pms1, PCNA, RFC-Δ1N, RPA, Mg^2+^, and Mn^2+^ were incubated at 30 °C for the indicated times. The formation of *Not*I-resistant products was monitored by the amount of unit length linear DNA formed after digesting products with *Sca*I and *Not*I. MW, molecular weight markers. (*C*) The average percent of *Not*I-resistant DNA from three independent experiments performed as in panel *B* was quantified as described in the legend to Fig. 1. (*D*) Reactions were performed as in panel B for 2 h at 30 °C with the indicated omissions and substitutions. (*E*) Reactions were performed as in panel B for 2 h at 30 °C with 5′ nicked substrates containing a +T, CC, AC, or TG mispair as indicated. (*F*) Reactions were performed as in panel B for 2 h at 30 °C and resistance to individual digestion by *Sac*I, *Nsp*I, *ApaI,* and *Not*I was determined as in panel C. We also measured excision of the 5′ nicked +T mispair substrate in the presence of Mg^2+^ and either 0.05 mM MnSO_4_ or ZnSO_4_ under these conditions and observed 10.5 ± 2.5% and 38 ± 4% resistance to *Apa*I digestion, respectively.

To further characterize gap formation, we digested the DNA products formed by Mlh1-Pms1 excision of the mispair-containing substrate with restriction endonucleases with recognition sites near the mispair to monitor conversion of cleavage-sensitive double-stranded DNA to cleavage-resistant DNA containing single-stranded gaps (32, 55) (Fig. 5*A*). We first found that the amount of *Not*I-resistant product DNA increased with increasing incubation time with Mlh1-Pms1 (Fig. 5 *B* and *C*). The formation of *Not*I-resistant product DNA was eliminated when: 1) Mlh1-Pms1 was omitted, 2) PCNA + RFC-Δ1N were omitted, 3) Mlh1-Pms1 was replaced by the Mlh1-Pms1(E707K) active site mutant (21, 23, 26, 38), or 4) Msh2-Msh6 was replaced by the Msh2-Msh6(F337A) mispair binding-defective mutant (32, 33, 50) (Fig. 5*D*), indicating requirements for Msh2-Msh6 mispair recognition and the endonuclease activity of Mlh1-Pms1. *Not*I-resistant product DNA was also formed in reactions containing substrates with a 5′ nick at the *Nae*I site and either +T, AC, TG, or CC mispairs (Fig. 5*E*). The formation of restriction endonuclease resistant product DNA was also observed with *Apa*I, *Nsp*I, and *Sac*I, which cut on either side of the mispair (Fig. 5*F*).

### Mlh1-Pms1 Catalyzes the Formation of a Broad Range of Single-Stranded Gap Sizes.

**Fig. 6.**
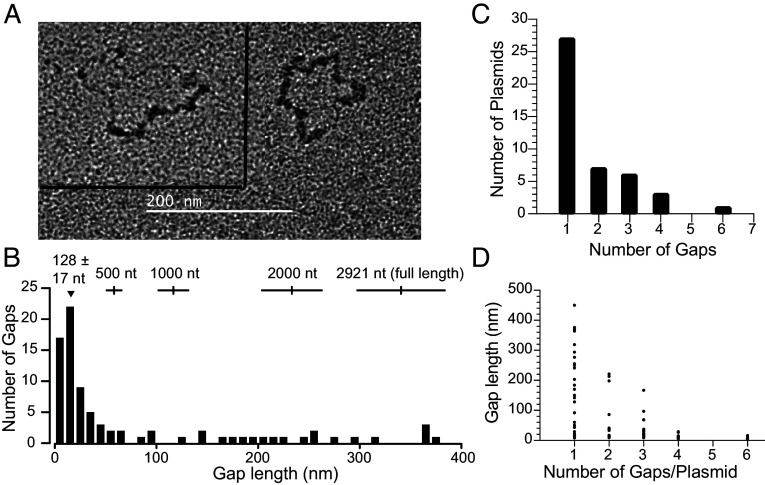
Electron microscopy shows that Mlh1-Pms1-catalyzed excision results in variable sized single-stranded gaps. An excision reaction containing a plasmid substrate with a +T mispair and a 5′ nick at the *Nae*I site, Msh2-Msh6, Mlh1-Pms1, PCNA, RFC-Δ1N, RPA, Mg^2+^, and Mn^2+^ was incubated at 30 °C for 2 h, the reaction products purified and the single-stranded DNA regions were stained with *Escherichia coli* SSB prior to visualization by electron microscopy. (*A*) Representative micrographs of products of the Mlh1-Pms1 excision reaction labeled with *E. coli* SSB in which single-stranded DNA regions appear thicker. (*B*) Histogram of the gap lengths (n = 83) observed in the micrographs of randomly selected DNA molecules. The scale factor for gap length in nm to gap length in nucleotides was determined by measuring the lengths of single-stranded circular control plasmids yielding a conversion factor of 1,000 nucleotides = 116.6 ± 14.7 nm. The peak of the gap sizes had a length of 128 ± 17 nucleotides, and most gaps were under 500 nucleotides. (*C*) Histogram of the number of gaps observed per plasmid. (*D*) The observed gap lengths as a function of the number of gaps in the plasmid. nt, nucleotides.

To gain insight into the single-strand gaps, we purified excision products from reactions that contained a +T mispair-containing plasmid substrate with a 5′ nick at the *Nae*I site, Msh2-Msh6, PCNA, RFC-Δ1N, and RPA, Mlh1-Pms1, Mg^2+^, and Mn^2+^. The single-stranded regions in the excision products were stained with *Escherichia coli* Single-Strand DNA Binding Protein (SSB) and visualized using electron microscopy (EM) (32) (Fig. 6*A*). Single-stranded gap sizes ranged from small gaps to gaps that were almost the size of the entire substrate (Fig. 6*B*). While most gapped products only contained a single gap, products containing 2 to 6 gaps were also observed (Fig. 6*C*); molecules with a single gap had an average gap length of 181 nm (range 7.8 to 449.8 nm, 1,553 nucleotides; Fig. 6*D*), whereas the gaps in the molecules with multiple gaps tended to be shorter with an average gap size of 37 nm (range 3.3 to 220.7 nm, 317 nucleotides; Fig. 6*D*). Note that this EM method cannot detect single-strand gaps sizes less than the length of an SSB tetramer binding site (30 nucleotides at 10 mM NaCl, 3.6 nm; see legend to Fig. 6) (56).

### Mlh1-Pms1 Generates Two Patterns of Single-Stranded Gaps On Plasmid Substrates.

We next used deamination by the single-strand DNA-specific cytidine deaminase APOBEC3A (57) to map the single-strand gaps produced by Mlh1-Pms1. We generated excision products in reactions containing a +T mispair-containing plasmid substrate with a 5′ nick at the *Nae*I site, Msh2-Msh6, PCNA, RFC-Δ1N, and RPA, Mlh1-Pms1, Mg^2+^, and Mn^2+^, cleaved them with *Sac*I to linearize DNA molecules without a gap, treated the excision products with APOBEC3A, and then filled in the gaps with T4 DNA polymerase and T4 DNA ligase. The resulting DNA molecules were transformed into an *ung* mutant *E. coli* strain, and plasmids from individual clones were sequenced to detect C > T transitions that occur only in single-stranded regions subject to deamination by APOBEC3A.

**Fig. 7.**
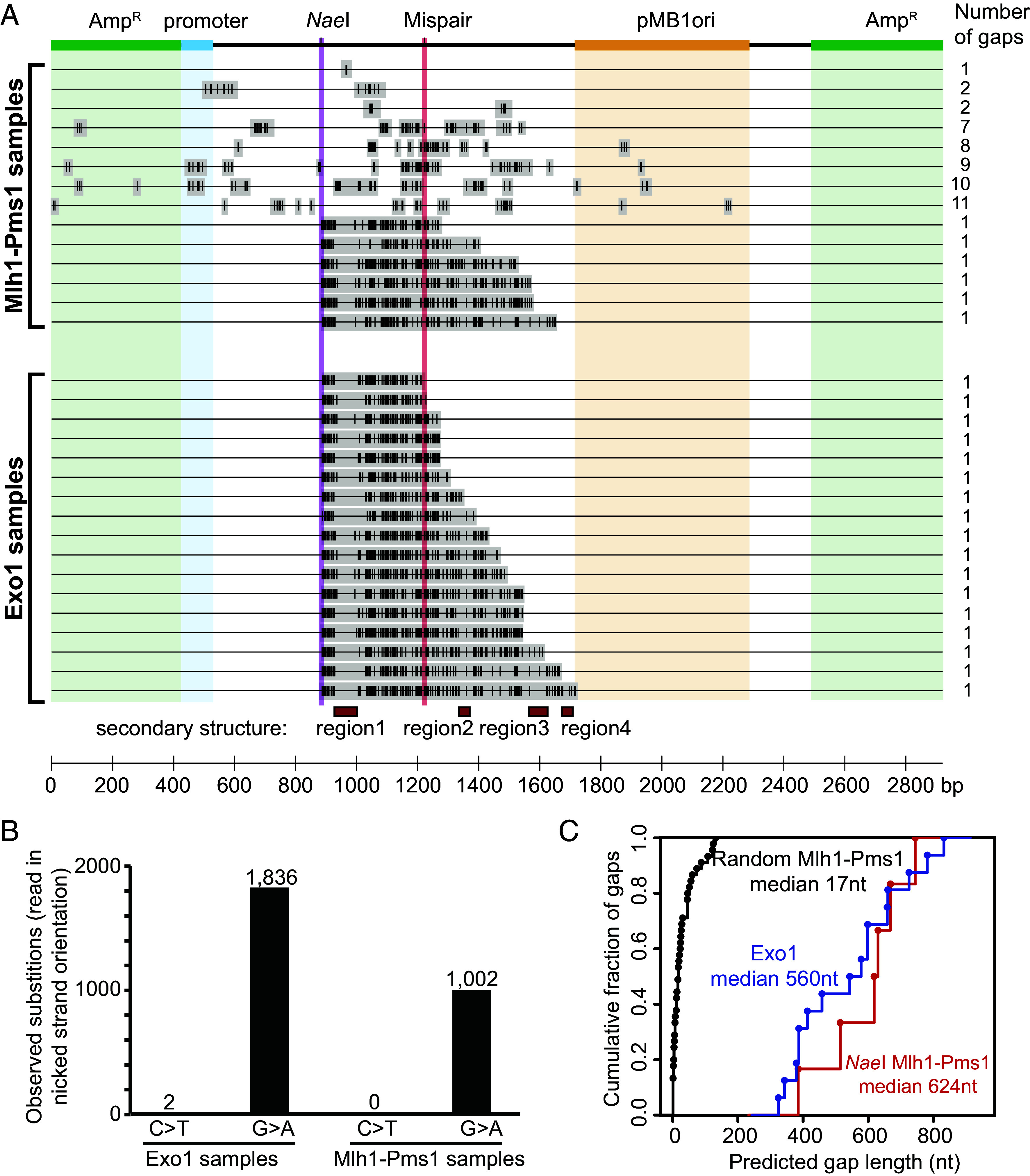
Cytidine deamination by APOBEC3A maps Mlh1-Pms1- and Exo1-catalyzed excision tracts. (*A*) In one experiment, repair reactions containing the 5′ nicked +T mispair substrate and Msh2-Msh6, Mlh1-Pms1, PCNA, RFC-Δ1N, RPA, Mg^2+^, and Mn^2+^ were incubated at 30 °C for 2 h and the product DNA was digested with *Sac*I. One aliquot of product DNA was incubated with T4 DNA polymerase followed by incubation with DNA ligase and a second aliquot of product DNA was incubated with APOBEC3A followed by incubation with T4 DNA polymerase and then by incubation with DNA ligase. In a control experiment, repair reactions containing the 5′ nicked +T mispair substrate (*SI Appendix*, Fig. S1) and Msh2-Msh6, Exo1, PCNA, RFC-Δ1N, RPA, and Mg^2+^ were incubated at 30 C for 2 h and the product DNA was digested with *Apa*I followed by sequential treatment with APOBEC3A, T4 DNA polymerase, and T4 DNA ligase. All three DNA samples were transformed into an *ung* mutant *E. coli* strain (BW310) and individual plasmids were subjected to whole plasmid sequencing. Deamination sites (vertical lines) and predicted gaps (gray bars) for individual cloned excision products from Mlh1-Pms1-only reactions and Exo1-only reactions are indicated on a map of the plasmid substrate. Regions of secondary structure that prevent deamination by APOBEC3A (*SI Appendix*, Fig. S3*C*) are shown as red boxes. (*B*) Observed deamination products in the nicked strand product are G > A substitutions indicating exclusive excision of the nicked strand followed by deamination and resynthesis. C > T substitutions would indicate excision of the continuous strand followed by deamination and resynthesis but none of these were recovered. (*C*) Gap lengths predicted for Mlh1-Pms1-catalyzed gaps at random sites, Mlh1-Pms1-catalyzed gaps initiating at the *Nae*I nick site and Exo1-catalyzed gaps initiating at the *Nae*I site nick; no other Exo1-catalyzed gaps were observed. nt, nucleotides.

Two types of control reactions were performed. 1) The first control omitted APOBEC3A treatment of the Mlh1-Pms1 excision products. Sequences of 31 plasmids recovered from these control reactions matched the reference plasmid, except in one clone where two base substitutions were observed. 2) The second control performed excision reactions containing Msh2-Msh6, PCNA, RFC-Δ1N, RPA, Exo1, Mg^2+^, but no Mn^2+^ or Mlh1-Pms1 (*Apa*I was also substituted for *Sac*I to linearize nongapped molecules) and included APOBEC3A treatment. Sequences of plasmids obtained from these Exo1 excision controls contained many C > T substitutions (Fig. 7 *A* and *B*), essentially all of which were consistent with deamination of the continuous strand of the substrate (G > A substitutions in the nicked strand orientation) and not the nicked strand (C > T substitutions in the nicked strand orientation). As expected from the 5′-to-3′ polarity of Exo1, deamination tracts from Exo1-treated samples began precisely at the *Nae*I nick site (Fig. 7*A* and *SI Appendix*, Fig. S4*A*) and extended in a 5′-to-3′ direction for 327 to 832 nucleotides (Fig. 7*C* and *SI Appendix*, Fig. S4*B*). Cytidines that were less frequently or never deaminated in the plasmids were in regions where the continuous strand was predicted to fold into a secondary structure (58) (*SI Appendix*, Fig. S4*C*) and/or recapitulated the preference of APOBEC3A for deaminating cytidines following a pyrimidine nucleotide more efficiently than cytidines following a purine (57, 59) (*SI Appendix*, Fig. S4*D*).

Like the Exo1 controls, all Mlh1-Pms1 deamination events were consistent with deamination of the continuous strand of the substrate (Fig. 7*B*). Unlike the Exo1 controls, two different deamination patterns were observed (Fig. 7*A*). Approximately ~60% of the plasmids had short and often multiple tracts of deamination (median length 17 nucleotides, range 1 to 132 nucleotides; Fig. 7*C*), consistent with the short single-strand gaps seen by EM. These deamination tracts did not start at the *Nae*I nick. The remaining ~40% of the plasmids had a single continuous deamination tract reminiscent of the Exo1 tracts; these tracts started at or near the *Nae*I nick and extended in a 5′-to-3′ direction for 385 to 767 nucleotides (Fig. 7 *A* and *C* and *SI Appendix*, Fig. S5*A*); the tracts showed the same deamination-resistant regions and APOBEC3A cytidine preferences as did the Exo1 tracts (*SI Appendix*, Fig. S5 *B* and *C*) although their median length was longer than the median length of the Exo1 tracks (Fig. 7*C*). These long tracts were reminiscent of the long single-stranded DNA gaps observed by EM but were not as long as the longest gaps seen by EM (Fig. 6); however, deamination of longer gaps would likely inactivate regions of the plasmid required for selection and propagation in *E. coli* resulting in plasmid loss.

## Discussion

Here we have biochemically reconstituted an Exo1- and Rad27-independent MMR reaction in which mispair excision is performed by the Mlh1-Pms1 endonuclease. Our results demonstrate the unexpected finding that the Mlh1-Pms1 endonuclease can excise mispairs by catalyzing the formation of strand-specific single-strand gaps on mispair-containing substrates in reactions that depend on mispair recognition and Mlh1-Pms1 loading by Msh2-Msh6 or Msh2-Msh3 and activation of the Mlh1-Pms1 endonuclease activity by RFC-loaded PCNA and Mn^2+^. Several lines of evidence argue for the critical role of gap formation catalyzed by Mlh1-Pms1 in these reactions: 1) the absolute requirement for Mlh1-Pms1, its endonuclease active site and its activation, 2) the ability of the nonstrand displacing DNA Polε to resynthesize the excised strand produced by Mlh1-Pms1 in reconstituted MMR reactions, 3) the ability of single-strand-specific Mung Bean Nuclease to cleave Mlh1-Pms1-catalyzed excision products, 4) the resistance of Mlh1-Pms1-catalyzed excision products to cleavage by restriction enzymes, which are double-strand DNA specific, 5) the direct observation of single-strand gaps in excision products by EM, and 6) the ability of APOBEC3A, a single-strand specific cytidine deaminase, to act on the single-strand DNA present in the gaps of the excision products. Consistent with a bona fide MMR reaction, our results on the polarity of repair and the polarity of the APOBEC3A cytidine deamination demonstrate that gap formation by Mlh1-Pms1 is strand-specific and targets the nicked strand in mispair-containing plasmid substrates allowing for strand-specific MMR.

Multiple lines of evidence have suggested the existence of an Mlh1-Pms1-catalyzed mispair excision pathway in MMR. Exo1 was the first excision nuclease implicated in Msh2-dependent MMR (15, 53); however, deletion of *EXO1* causes only a small MMR defect in *Saccharomyces cerevisiae* that is less that 1% of the MMR defect caused by deletion of *MSH2*, and *Exo1^−/−^* knockout mice have far less severe cancer phenotypes than *Msh2^−/−^* or *Mlh1^−/−^* mice (15, 41, 60). These results argue that other redundant MMR excision pathways exist and led to a genetic screen in *S. cerevisiae* to identify *exo1Δ*-synergizing mutations affecting these redundant MMR pathways and additional studies of candidate mutations. Mutations affecting these other pathways, which synergize with *exo1Δ* and *exo1* truncation mutations (18, 19, 26, 41, 43, 51, 61), delineate two alternative excision pathways. The first group of mutations, including *pol32-Q46stp* and *rad27Δ*, cause defects in the Rad27-dependent excision pathway. This pathway depends on 5′ flaps generated through strand-displacement synthesis by DNA Polδ (33), which requires the Pol32 subunit for forming long flaps (62) and is coordinated by Msh2-Msh6 or Msh2-Msh3. The second group of mutations, including those affecting PCNA and Mlh1-Pms1, cause defects in an Mlh1-Pms1 endonuclease activation pathway, and include 1) mutations affecting the Mlh1-Pms1 ATPase active sites, 2) mutations affecting interactions between Mlh1 and Pms1, 3) mutations affecting the region of Pms1 that interacts with PCNA, and 4) mutations affecting different biochemical features of PCNA. Notably, none of the *exo1Δ*-synergizing mutations completely eliminate Mlh1-Pms1 or its endonuclease activity, which was interpreted as suggesting that Exo1-independent MMR requires higher levels of Mlh1-Pms1 endonuclease activity than Exo1-dependent MMR (26, 38, 43). An alternative screen for dominant *pms1* mutations identified most of the ligands for the Pms1 active site metals (26). These dominant mutants cause a weak MMR defect in a wild-type strain and a strong MMR defect in an *exo1Δ* strain, suggesting that one of the Exo1-independent excision pathways is exquisitely sensitive to the presence of a mixture of wild-type and endonuclease-defective Mlh1-Pms1 complexes, consistent with the results with *exo1Δ*-synergizing mutations.

Mlh1-Pms1 recruited to mispair-containing substrates containing a nicked strand and activated by RFC-loaded PCNA is known to introduce a nick or nicks into the already nicked strand to promote mispair excision through 5′-to-3′ excision by Exo1 (21, 23, 38), and presumably also by Rad27 and DNA Polδ (33). Here, we have shown that this recruited and activated Mlh1-Pms1 also catalyzes the formation of nicked strand-specific gaps that result in mispair excision. The generation of closely spaced nicks would possibly produce unstable oligonucleotides products that dissociate from the continuous strand thus resulting in gap formation. This idea appears consistent with a recent report that ATP hydrolysis allows Mlh1-Pms1 to turnover from its product nick allowing it to make additional nicks (63) and would explain the fact that Mlh1-Pms1 ATP hydrolysis mutations cause synergistic MMR defects when combined an *exo1Δ* mutation (41, 61). In addition, studies mapping single human Mlh1-Pms2 (Mlh1-Pms1 in yeast)-catalyzed nicks in mispair-containing regions have observed nicked sites (64), that if occurring in pairs, could be in the range of 10 to 20 nucleotides apart. The smallest deamination tracts observed here contain only single deamination sites, which would be consistent with Mlh1-Pms1-catalyzed adjacent nicks being very close together (<10 nucleotides) assuming efficient deamination by APOBEC3A. Oligonucleotides in these size ranges could either dissociate on their own or in the presence of nick-binding (PCNA and RFC) or ssDNA binding (RPA) proteins under physiological conditions.

A striking finding of our studies is the formation of two classes of Mlh1-Pms1-catalyzed single-stranded gaps including single long polar gaps that appear to initiate at a preexisting nick and multiple dispersed small gaps. These could reflect different types of DNA-bound Mlh1-Pms1 sliding clamps. The dispersed gaps could reflect a more distributive Mlh1-Pms1-PCNA clamp complex, a Msh2-Msh6-Mlh1-Pms1 sliding clamp complex or Mlh1-Pms1 sliding clamps lacking any other MMR proteins (14, 65, 66). The nature of the Mlh1-Pms1 complexes underlying the polar gaps that appear to initiate at the *Nae*I nick are unclear and could be consistent with several possibilities. 1) Mlh1-Pms1 bound to RFC and PCNA that load at the nick could result in a processive activated endonuclease complex, where the apparent 5′-to-3′ excision polarity could reflect the rebinding of RFC to new 3′ ends generated by Mlh1-Pms1 activity (67). 2) Mlh1-Pms1 complexed with Msh2-Msh6 has different diffusion dynamics in single molecule experiments than Mlh1-Pms1 alone, and further Msh2-Msh6 complexes with Mlh1-Pms1 have also been observed to form higher-order assemblies by atomic force microscopy (68) which could underlie polar excision. 3) Recruitment of multiple Mlh1-Pms1 complexes, consistent with the ~11 molecules present in Pms1 foci in vivo (18), could form higher-order complexes, which has been observed for Mlh1-Pms1 and Mlh1-Mlh3 (69, 70), or could exhibit an apparent 5′-to-3′ excision polarity due to steric occlusion with an adjacent product nick-bound complex.

**Fig. 8.**
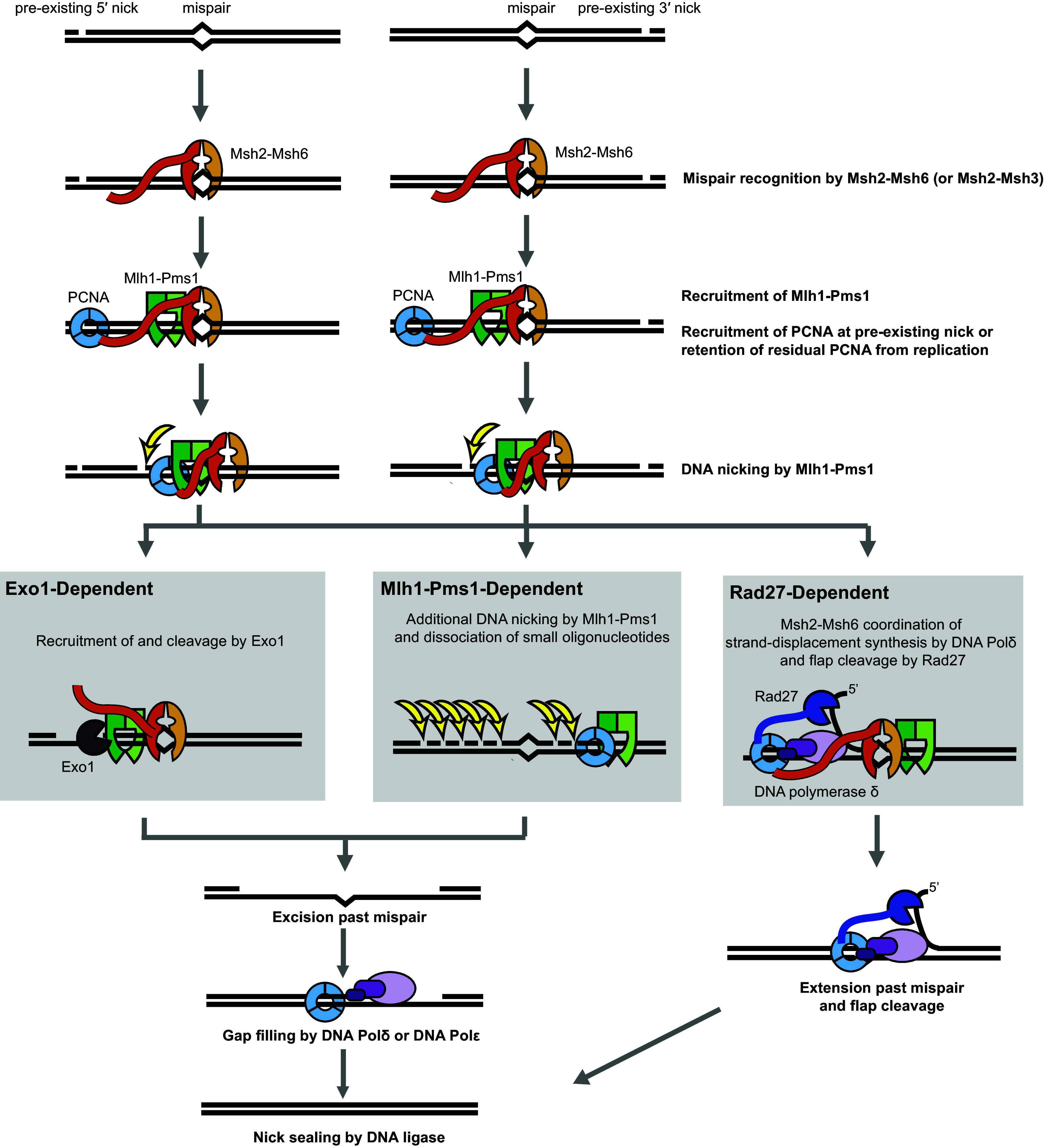
Model for the three MMR excision pathways. MMR is initiated when Msh2-Msh6 (or Msh2-Msh3) recognizes a mispair, exchanges ADP for ATP, and recruits Mlh1-Pms1 and interacts with PCNA. Excision of the nicked strand occurs by one of three redundant pathways. In the Exo1-dependent pathway, Exo1 is recruited by Msh2-Msh6 and/or Mlh1-Pms1 to a nick 5′ of the mispair allows for 5′-to-3′ resection of the nicked strand past the mispair to generate a gap that can be filled by DNA Polδ or DNA Polε. In the Rad27-dependent pathway, strand-displacement synthesis starting at a nick 5′ of the mispair by DNA Polδ and 5′ flap cleavage by Rad27 is coordinated by Msh2-Msh6, likely through shared interactions with PCNA. In both pathways, 5′ nick can be preexisting or could be made by the Mlh1-Pms1 endonuclease. In the Mlh1-Pms1-dependent excision pathway, additional rounds of nicking generate gaps that can be filled by DNA Polδ or DNA Polε. In each reaction, polarity of the repair is controlled directly by the preexisting nick or indirectly by the polarity of the Mlh1-Pms1 endonuclease, which only nicks strands containing the preexisting nicks.

Biochemical reconstitution of the Mlh1-Pms1 excision pathway allows a number of key redundant MMR excision pathways to be defined (Fig. 8). MMR is initiated when Msh2-Msh6 or Msh2-Msh3 recognize a mispair in DNA, exchange ADP for ATP, and recruit Mlh1-Pms1. When the mispair-containing DNA also contains a nick and Mlh1-Pms1 is activated by RFC-loaded PCNA, Mlh1-Pms1 introduces an additional nick or nicks into the already nicked strand. This nicking is proposed to maintain strand-specific nicks to ensure the polarity of the excision reactions and may be the essential role of the Mlh1-Pms1 endonuclease in all MMR pathways (39, 42). In the Exo1 excision pathway, Exo1 is recruited by Msh2-Msh6 and/or Mlh1-Pms1 through its MIP and SHIP boxes (19, 24, 25). When recruited to a preexisting or Mlh1-Pms1-generated nick 5′ of the mispair, the Exo1 5′-to-3′ exonuclease activity excises the mispair and leaves a gap. In the Rad27 excision pathway, recruitment of DNA Polδ to a nick 5′ of the mispair, which may be facilitated by the interaction of PCNA with Msh6 and Msh3, leads to strand-displacement synthesis. This repairs the mispair and generates a 5′ flap that is cleaved by Rad27 in a reaction that is likely coupled to DNA Polδ and PCNA. In the Mlh1-Pms1 excision pathway, repetitive nicking by PCNA-activated Mlh1-Pms1 generates gaps, presumably requiring its ATPase activity to dissociate from products in each nicking cycle (63). The gaps can then be filled by either DNA Polδ or DNA Polε, and the remaining single-stranded nick is ligated. Together, these three excision pathways account for most or all of MMR, as combining a *rad27Δ* mutation, an MMR-defective *exo1* truncation, and *mlh1-A99V*, which affects the ATPase domain, causes a mutator phenotype that is close to that caused by deletion of *MSH2* and other essential MMR genes (33).

## Materials and Methods

### Proteins.

*S. cerevisiae* Msh2-Msh6, Msh2-Msh3, Mlh1-Pms1, PCNA, RFC-Δ1N, RPA, Exo1, DNA Polδ, DNA Polε as well as mutant proteins were all overexpressed and purified as described previously (31, 32, 38). Multiple batches of Msh2-Msh6, Mlh1-Pms1, and DNA polymerase ε were used and in some cases the protein preparations were also used in previously published experiments (31, 32, 38, 71). All protein preparations were >95% pure. Restriction endonucleases, Mung Bean Nuclease, T4 DNA polymerase, and T4 DNA ligase were obtained from New England Biolabs (NEB) and used with reaction buffers provided by the manufacturer. *E. coli* DNA Gyrase was obtained from Sigma-Aldrich. APOBEC3A was provided by Dr. Reuben Harris from the University of Texas Health Sciences Center at San Antonio.

### Construction of DNA Substrates.

The plasmid DNA substrates containing a CC, AC, TG, +T, +TGC, or +ACGC mispair and either a 5′ nick at the *Nae*I site or a 3′ nick at the *Afl*III site were constructed using mutant pBluescript plasmids (*SI Appendix*, Fig. S1) as previously described with one modification (32). Instead of being purified by chromatography on a BND cellulose column, the substrate DNAs were purified by HPLC. The DNAs were bound to a GenPac FAX column (Waters) followed by elution with a NaCl gradient from 100 mM (Buffer A) to 1 M NaCl (Buffer B) in 10 mM Tris pH 8.0, 1 mM EDTA buffer; the gradient was run at 0.5 mL per min with an increase in Buffer B of 1% per min. After evaluating purity by agarose gel electrophoresis, some batches of substrate were subjected to a second round of purification. Finally, the substrate DNAs were concentrated by ethanol precipitation and were suspended in 10 mM Tris, pH 8.0, 1 mM EDTA buffer. The substrate with the defined 26 nucleotide gap was constructed and purified using essentially the same method as the mispair-containing substrates described above, except that the pRDK1253 discontinuous strand donor was digested with both *Kpn*I and *Bam*HI prior to addition to continuous single-stranded DNA circles derived from pRDK1252.

### In Vitro MMR Assays.

MMR assays were performed by a modification of our previously published procedures (31–33, 38). Briefly, proteins were diluted in 7.5 mM HEPES, pH 7.5, 10% (vol/vol) glycerol, 200 mM KCl, 1 mM DTT, and 0.5 mg/mL BSA. To set up a standard 10 μL reaction, 390 fmol of Mlh1-Pms1, 390 fmol Msh2-Msh6, 290 fmol of PCNA (PCNA trimers), 400 fmol of DNA Polε, 220 fmol of RFC-Δ1N, and 1,800 fmol of RPA were combined into a total volume of 4 μL. As indicated in some experiments, 0.38 fmol of Exo1 was added, 80 fmol of DNA Polδ was substituted for DNA Polε, and individual proteins were omitted, and mutant proteins were substituted for wild-type proteins. Then 1 μL of 100 ng/μL of substrate DNA and 5 μL of 33 mM Tris pH 7.6, 75 mM KCl, 2.5 mM ATP, 1.66 mM glutathione, 8.3 mM MgCl_2_, 1 mM MnSO_4_, 80 μg/mL of BSA, and 200 μM each of the dNTPs were added. Unless otherwise indicated, reactions were incubated at 30 °C for 2 h in reactions without Exo1 and 3 h in reactions containing Exo1. The reactions were terminated by the addition of 500 mM EDTA to a concentration of 20 mM, 20 μL of 360 μg/mL of proteinase K and a final concentration of 0.4 mg/mL of glycogen and incubated at 55 °C for 30 min. The DNA in each reaction was purified by phenol extraction and ethanol precipitation, digested with 5 units of *Sca*I and 2 units of *Pst*I (or *Xho*I, *Nsi*I, or *Nsp*I as indicated in specific experiments; see Fig. 3) in NEB rCutSmart buffer for 1 h at 37 °C and analyzed by agarose gel electrophoresis as described under “Cleavage analysis”.

### Cleavage Analysis.

EthBr stained gels were photographed and quantified using a BioRad ChemiDoc MP imaging system and Image Lab software, version 4.1. The amount of DNA in the repair-specific 1.1-kb and 1.8-kb fragments was then expressed as the percent of total DNA present in the 1.1-kb and 1.8-kb and 2.9-kb (substrate) fragments. In all repair assays, 100% repair is 100 ng or 52.75 fmol of substrate. In experiments where the results of multiple independent experiments were quantified and the results presented as histograms, the bars are the average values, the whiskers indicate the SEM and the dots indicate the values from (n ≥ 3) independent reactions.

### Excision Assays.

Mlh1-Pms1-catalyzed excision reactions lacked DNA polymerases and were otherwise performed as described for the in vitro MMR assays and processed through the phenol extraction and ethanol precipitation step. The excision reactions were incubated for 2 h at 30 °C unless otherwise indicated. Exo1-catalyzed excision reactions were similarly performed except that the reactions 1) contained 0.38 fmol Exo1, 2) Mlh1-Pms1, DNA polymerases, and Mn^2+^ were omitted, and 3) reactions were incubated for 3 h at 30 °C. The reaction products were then either digested with Mung Bean Nuclease or digested with *Sca*I to linearize the DNA and one of *Apa*I, *Not*I, *Sac*I, or *Nsp*I that cleave the plasmid substrates near the mispair as indicated in individual experiments. Digestions were performed in 10 μL reactions containing 100 ng DNA, 1× NEB rCutSmart buffer, and 2 to 5 units of nuclease as appropriate for 1 h at 37 °C and the DNA products were analyzed by agarose gel electrophoresis as described under “Cleavage analysis.”

### *Pst*I Oligonucleotide Cleavage Assays.

We constructed 60 bp long oligonucleotide duplex DNA substrates containing complete or partial *Pst*I cleavage sites (*SI Appendix*, Fig. S3) by annealing combinations of 2 or 3 oligonucleotides (*SI Appendix*, Tables S1 and S2). Annealing mixtures contained 1.97 pMoles of each oligonucleotide in 80 μL of annealing buffer (200 mM Tris HCI, pH 8.0, 4 mM EDTA, 200 mM NaCl) and were first heated in a heat block at 85 °C for 5 min followed by transferring the samples to room temperature and allowing them to cool to room temperature over approximately 1 to 2 h. Then 100 ng of each substrate was digested with 20 units of *Pst*I in a final volume of 10 μL of 1× NEB rCutSmart buffer for 1 h at 37 C followed by electrophoresis through 20% acrylamide TBE gels (Invitrogen) that were quantified as described under “Cleavage analysis”.

### *Pst*I Plasmid Cleavage Assays.

Covalently closed supercoiled DNA substrates were generated by incubating *Nae*I nicked plasmid substrates containing either a +T or CC mispair (*SI Appendix*, Fig. S1) with T4 DNA ligase and *E. coli* DNA gyrase as previously described (33). Then 100 ng of each substrate was digested with 2 units of *Pst*I in a final volume of 10 μL of 1× NEB rCutSmart buffer for 1 h at 37 C followed by agarose gel electrophoresis and quantitation as described under “Cleavage analysis.”

### APOBEC3A-Mediated Deamination Analysis.

Six Mlh1-Pms1 excision reactions (100 ng substrate per reaction) were performed as described under the “Excision assays” and the 600 ng of product DNA was pooled to generate an “Mlh1-Pms1 treated” sample. Six Exo1 excision reactions were similarly performed and pooled to generate an “Exo1 treated” sample. 100 ng of product from each sample was used to determine the fraction of DNA that had been excised by performing a *Sac*I/*Sca*I (Mlh1-Pms1 sample) or an *Apa*I/*Sca*I (Exo1 sample) double digest and monitoring the fraction of substrate that was only linearized. The remaining 500 ng of each sample was then digested with *Sac*I (Mlh1-Pms1 sample) or *Apa*I (Exo1 sample) to select against plasmids that were double-stranded in these regions. 100 ng of the 500 ng of digested product from each sample was used in a APOBEC3A-minus control and incubated in a 10 μL reaction containing NEB buffer r2.1, 10 mM ATP, 100 mM DTT, 250 μM each of the dNTPs and 0.6 units of T4 DNA polymerase (New England Biolabs) for 30 min at 37 °C followed by addition of 0.38 units of T4 DNA ligase (New England Biolabs) and further incubated for 30 min at 37 °C. To the remaining 400 ng of the 500 ng of digested product, 0.5 M EDTA was added to a final concentration of 10 mM, and the product DNA was incubated in four 10 μL reactions containing 20 mM Tris pH 6.5, 0.1% Tween 20, 10% DMSO, and 140 nM APOBEC3A for 1 h at 25 °C after which the DNA was purified by phenol extraction and ethanol precipitation and then treated with T4 DNA polymerase and T4 DNA ligase as indicated for the APOBEC3A-minus samples. Aliquots of the DNA samples were then used to transform the *ung* mutant *E. coli* strain BW310 (Hfr (PO-45) *relA1 spoT1 thi-1 ung-1*) that was provided by Dr. Reuben Harris. Plasmid DNA from individual transformants was purified using standard methods and subjected to whole plasmid sequencing (Plasmidsaurus). Plasmid sequences were deposited in the Zenodo data repository (72).

For excision tracts initiating at the *Nae*I site, all sequence changes were identified for each plasmid recovered. Except for two nicked strand deamination events (C > T substitutions) in one Exo1-excised sample, all changes were continuous strand deamination events (G > A substitutions), which were plotted by sample. For the Exo1-excised samples and the Mlh1-Pms1-excised samples with excision tracts initiating at the *Nae*I site, sample-specific deamination tracts spanning from the first to the last observed deamination event could be defined. These tracts allowed the determination of the fraction of observed deamination events at each position and the fraction of cytidine-containing dinucleotides on the continuous strand (A-C, T-C, G-C, C-C, C-A, C-T, C-C, and C-G) that were deaminated. To determine the position-dependent fraction of deamination, the fraction of observed deamination events at each position were determined using samples whose deamination tracts spanned that position. To determine the fraction of deamination of each type of dinucleotide, the deaminated and unmodified dinucleotides were counted within the observed deamination tracts after excluding regions containing secondary structures identified by UNAFOLD (58).

For Mlh1-Pms1 excised samples with short random excision tracts, initial excision tracts were assigned by identifying regions where every continuous strand cytidine was deaminated. Adjacent tracts were merged if they were separated by a small number of cytidines that were not deaminated (median of 2 nondeaminated cytidines); these cytidines tended to be in purine-cytidine dinucleotides (A-C and G-C, 116 cases) rather than pyrimidine-cytidine dinucleotides (C-C and T-C, 34 cases), consistent with the substrate preference of APOBEC3A (57, 59).

### Electron Microscopy.

Products of Mlh1-Pms1-catalyzed excision were analyzed by electron microscopy after staining of single-strand DNA regions with *E. coli* SSB as previously described (32) with the following modifications: 1) Four 10 μL excision reactions containing the 5′ nicked +T mispair substrate and lacking DNA Polε were incubated at 30 °C for 2 h and pooled; 2) Glycogen was omitted; and 3) the DNA was suspended in 20 μL of 10 mM HEPES, pH 7.5, 0.1 mM EDTA prior to staining with *E. coli* SSB and mounting for electron microscopy which included fixation with 0.6% glutaraldehyde for 5 min at room temperature, dehydration through an ethanol series and rotary shadow casting with tungsten. The lengths of DNA molecules were measured using ImageJ software.

## Supplementary Material

Appendix 01 (PDF)

## Data Availability

DNA Sequence Files data have been deposited in Zendo Data Repository (https://doi.org/10.5281/zenodo.17107465) (72). All study data are included in the article and/or *SI Appendix*.
