# Supplementary material for: DNA mismatch repair mediated by Mlh1–Pms1 endonuclease-catalyzed mispair excision
Source: Proc Natl Acad Sci U S A. 2025 Dec 24;122(52):e2528670122. doi: 10.1073/pnas.2528670122 (PMC12772206; doi:10.1073/pnas.2528670122)
Supplement: Supplementary file 1 — Appendix 01 (PDF) [file pnas.2528670122.sapp.pdf]

SUPPLEMENTARY INFORMATION FOR

DNA mismatch repair mediated by Mlh1-Pms1 endonuclease-catalyzed mispair excision

Tatiana Palacio[a,1], Felipe A. Calil[a,1,2], Nikki Bowen[a], Jack D. Griffith[b,c], Christopher D. Putnam[d,e] and Richard D. Kolodner[a,e,f,3]

[a] Department of Cellular and Molecular Medicine, University of California San Diego School of Medicine, La Jolla, CA 92093-0660, USA

[b] Program in Virology, Lineberger Cancer Center, University of North Carolina at Chapel Hill, Chapel Hill, NC 27514

[c] Department of Microbiology and Immunology, University of North Carolina at Chapel Hill, Chapel Hill, NC 27514

[d] Department of Pediatrics, University of California San Diego School of Medicine, La Jolla, CA 92093-0660, USA

[e] Structural and Functional Genomics Program, Moores–University of California San Diego Cancer Center, University of California San Diego School of Medicine, La Jolla, CA 92093-0660, USA

[f] Institute of Genomic Medicine, University of California San Diego School of Medicine, La Jolla,
CA 92093-0660, USA

Running head: Endonuclease-mediated mismatch repair

[1] Co-first authors

[2] Present address: Department of Biochemistry, Institute of Chemistry, University of São Paulo, 05508-0000 São Paulo, Brazil

[3] To whom correspondence should be addressed:
Tel: 858-534-7804; Email: rkolodner@health.ucsd.edu

A

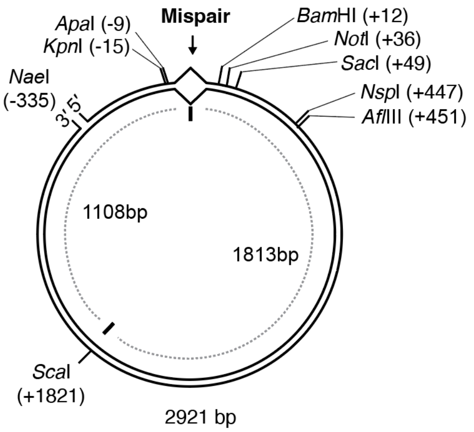

B

Mispair	Mispaired substrate	Donor Plasmids
TG	*Apa*I *Nsi*I *Bam*HI<br>5′–...GGGCCCAATATGCATTCGAGGCGGGATCC...–3′<br>3′–...CCCGGGTTATACGTGAGCTCCGCCCTAGG...–5′<br>*Xho*I	pRDK506<br>pRDK505
AC	*Apa*I *Xho*I *Bam*HI<br>5′–...GGGCCCAATATGCACTCGAGGCGGGATCC...–3′<br>3′–...CCCGGGTTATACGTAAGCTCCGCCCTAGG...–5′<br>*Nsi*I	pRDK505<br>pRDK506
CC	*Apa*I *Xho*I *Bam*HI<br>5′–...GGGCCCAATCTGCACTCGAGCGGGATCC...–3′<br>3′–...CCCGGGTTAGACGTCAGCTCGCCCTAGG...–5′<br>*Pst*I	pRDK507<br>pRDK508
+T	*Apa*I *Nsp*I *Bam*HI<br>5′–...GGGCCCAATCTGCATGTCGAGGCGGGATCC...–3′<br>3′–...CCCGGGTTAGACGT–CAGCTCCGCCCTAGG...–5′<br>*Pst*I	pRDK1253<br>pRDK1252
+ACG	*Apa*I *Aat*II *Bam*HI<br>5′–...GGGCCCAATCTGCAGACGTCGAGGCGGGATCC...–3′<br>3′–...CCCGGGTTAGACGTC–––AGCTCCGCCCTAGG...–5′<br>*Pst*I	pRDK514<br>pRDK508
+ACGC	*Apa*I *Mlu*I *Bam*HI<br>5′–...GGGCCCAATCTGCAACGCGTCGAGGCGGGATCC...–3′<br>3′–...CCCGGGTTAGACGT––––CAGCTCCGCCCTAGG...–5′<br>*Pst*I	pRDK513<br>pRDK508

Supplemental Figure 1. Map and linker sequences of mispair-containing plasmid substrates. (A) Map of the plasmid-based substrate showing the positions of the mispair, the *Nae*I site 5′ nick, the *Afl*III site 3′ nick, the *Sca*I site used in MMR assays, the *Apa*I and *Bam*HI sites between which the mispair-containing linker sequences were inserted, and the *Apa*I, *Not*I, *Sac*I and *Nsp*I sites used in excision assays. **(B)** Sequences of the mispair-containing linker regions of the plasmid-based substrates. The top strand is the nicked strand, and the bottom strand is the continuous strand in the plasmid substrates. The *Apa*I and *Bam*HI cloning sites are indicated as are the relevant mispair (yellow) and the top and bottom strand restriction sites that are disrupted by each mispair. Plasmids that donate the top and bottom strands are indicated. Note that the +ACG insertion can exist in a second conformation that still disrupts the same *Aat*II and *Pst*I sites.

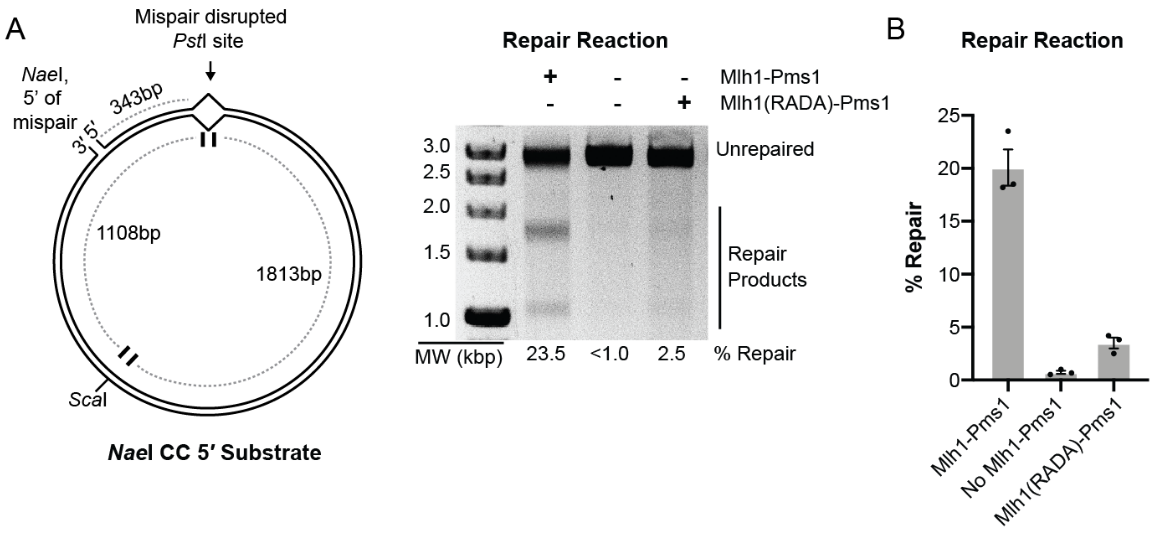

Supplemental Figure 2. Mutation of the conserved Mlh1 linker motif that promotes endonuclease activity eliminates Mlh1-Pms1-dependent MMR. (A) Map of the plasmid-based substrate showing the positions of the +T mispair that disrupts a *Pst*I site in the continuous strand (**Fig. S1**), the *Nae*I site 5′ nick and the *Sca*I site. **(B)** Representative assays of 5′ nick-directed repair of the +T substrate, in reactions containing Msh2-Msh6, Mlh1-Pms1, PCNA, RFC-Δ1N, RPA, DNA Polε, Mg^{2+} and Mn^{2+} in which Mlh1-Pms1 was included, omitted, or replaced with the Mlh1-R401A-D403A-Pms1 linker mutant complex as indicated that were incubated at 30 °C for 3 hr. Repair products were detected and quantified as described in the legend to **Fig. 1**. MW, molecular weight markers. **(C)** The average value for the percent of substrate repaired was quantified as described in the legend to **Fig. 1**. MW, molecular weight markers.

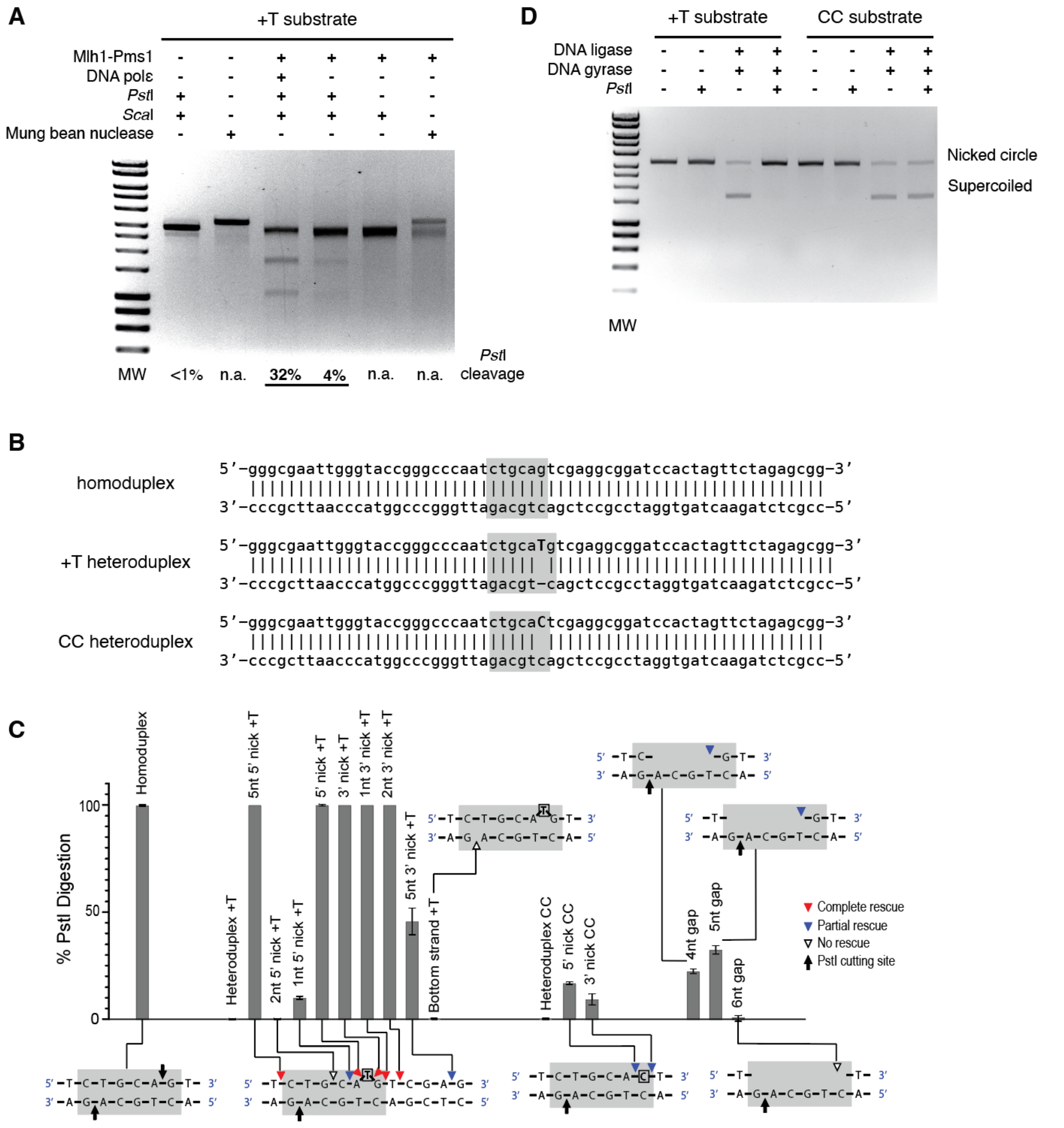

Supplemental Figure 3. Nicks and short gaps adjacent to the +T or CC mispairs restores *Pst*I cleavage of mispair-containing substrates. (A) Repair reactions containing the 5′ nicked +T mispair substrate and Msh2-Msh6, Mlh1-Pms1, PCNA, RFC-Δ1N, RPA, DNA Polε, Mg^{2+} and Mn^{2+} in which DNA Polε and Mlh1-Pms1 were present or absent as indicated were incubated at 30 °C for 3 hr. Repair was detected by digestion with *Sca*I and *Pst*I and single stranded gap formation was detected by digestion with Mung Bean Nuclease as indicated

followed by analysis by agarose gel electrophoresis. MW, molecular weight markers. In the complete reaction, digestion by *Sca*I and *Pst*I generated diagnostic 1.8 and 1.1 kbp bands indicating 32% of the DNA was repaired to be sensitive to *Pst*I cleavage. When DNA Polε was omitted, apparent repair was observed at a much lower, 4%, level and a fraction of the product DNA was also sensitive to cleavage by Mung Bean Nuclease; also see **Fig. 4**. MW, molecular weight markers; n.a., not applicable. **(B)** Sequences of the intact parental homoduplex, +T-mispair containing heteroduplex, and CC-mispair containing heteroduplex substrates generated by annealing oligonucleotides (**Tables S1, S2**); nicks and gaps introduced by annealing appropriate oligonucleotides are shown in panel C. **(C)** The indicated oligonucleotide duplexes were digested with *Pst*I, the products fractionated by acrylamide gel electrophoresis and the percent of DNA cleaved was quantified as described in the legend to **Fig. 1**. **(D)** 5′ nicked +T and CC mispair-containing substrates were treated with T4 DNA ligase and *E. coli* DNA gyrase to generate supercoiled mispair-containing plasmid substrates as indicated. The DNAs were digested with *Pst*I and cleavage was detected by analysis by agarose gel electrophoresis. In the absence of pretreatment by ligase and gyrase, the substrates were nicked circles and were resistant to double-stranded cleavage by *Pst*I because of the +T and CC mispairs. Ligase and gyrase pretreatment covalently closes and supercoils a subset of the substrate DNA molecules. *Pst*I digestion of the supercoiled +T mispair containing substrate but not CC mispair containing substrate efficiently converted it to a nicked circular form, indicating single strand nicking of the +T mispair containing substrate by *Pst*I.

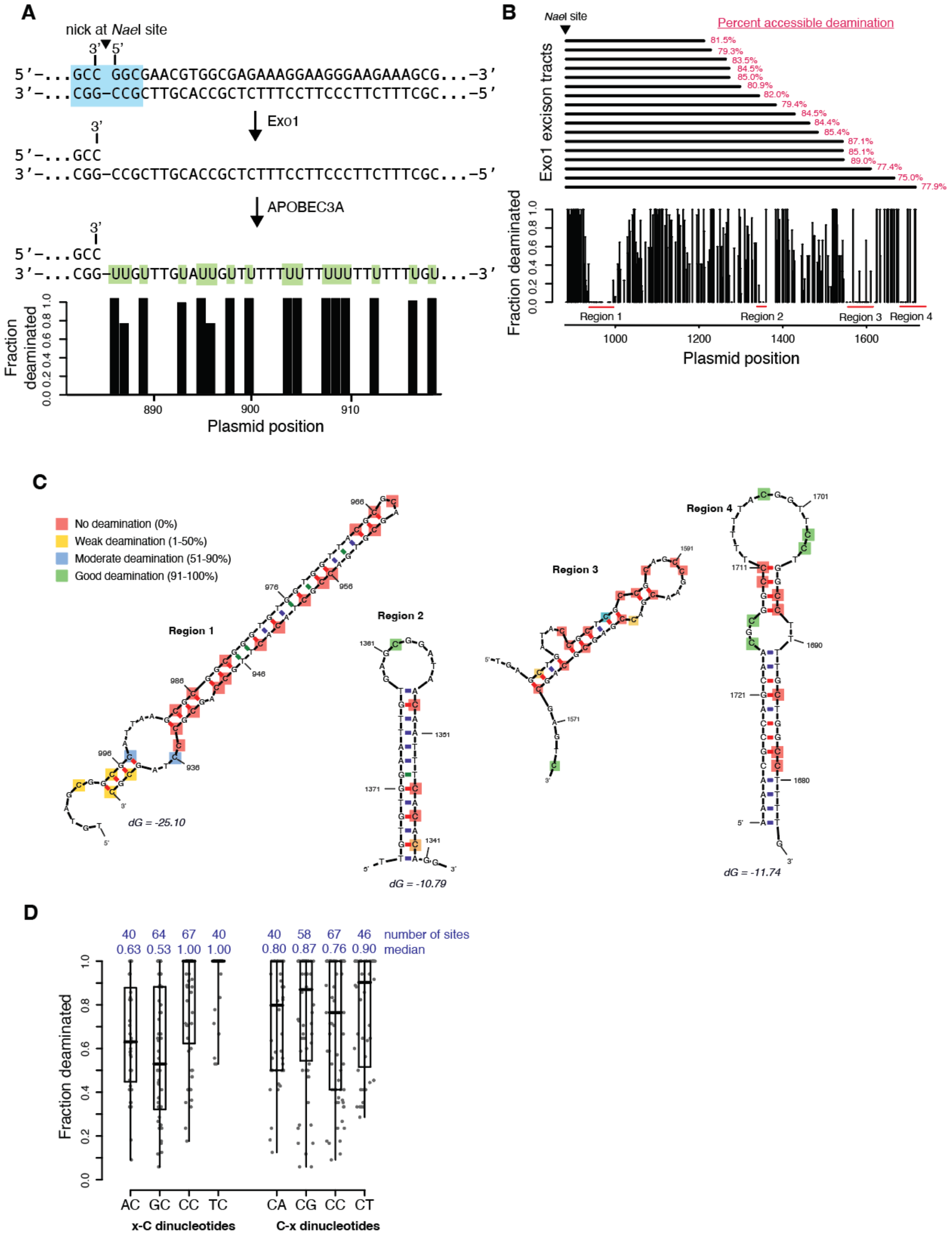
A
nick at NaeI site
3' 5'
5'–...GCC GGCGAACGTGGCGAGAAAGGAAGGGAAGAAAGCG...–3'
3'–...CGG–CCGCTTGCACCGCTCTTTCCTTCCCTTCTTTCGC...–5'
Exo1
3'
5'–...GCC
3'–...CGG–CCGCTTGCACCGCTCTTTCCTTCCCTTCTTTCGC...–5'
APOBEC3A
3'
5'–...GCC
3'–...CGG–UUGUTTGUAUUGUTUTTTUUTTUUUTTUTTTUGU...–3'
Fraction deaminated
0.0 0.2 0.4 0.6 0.8 1.0
890
900
910
Plasmid position
B
NaeI site
Percent accessible deamination
Exo1 excison tracts
81.5%
79.3%
83.5%
84.5%
85.0%
80.9%
82.0%
79.4%
84.5%
84.4%
85.4%
87.1%
85.1%
89.0%
77.4%
75.0%
77.9%
Fraction deaminated
0.0 0.2 0.4 0.6 0.8 1.0
Region 1
Region 2
Region 3
Region 4
1000
1200
1400
1600
Plasmid position
C
No deamination (0%)
Weak deamination (1-50%)
Moderate deamination (51-90%)
Good deamination (91-100%)
Region 1
dG = -25.10
Region 2
dG = -10.79
Region 3
Region 4
dG = -11.74
D
40 64 67 40 40 58 67 46 number of sites
0.63 0.53 1.00 1.00 0.80 0.87 0.76 0.90 median
Fraction deaminated
0.0 0.2 0.4 0.6 0.8 1.0
AC GC CC TC CA CG CC CT
x-C dinucleotides
C-x dinucleotides

Supplemental Figure 4. APOBEC3A-mediated deamination tracts of products from the Exo1 excision reaction. Exo1-generated excision products were generated with the 5′ N*ae*I site nicked +T mispair substrate, deaminated with APOBEC3A, and analyzed by DNA sequencing as indicated in Fig. 7. **(A)** Exo1 excision of the nicked strand and APOBEC3A-mediated cytidine deamination efficiently converted cytidines adjacent to the *Nae*I nick site to uracils as measured by G>A substitutions (when read on the nicked strand). The extent of deamination was calculated as the fraction of times the base was observed as deaminated in independent plasmid isolates. Analysis of the first 34 nucleotides starting from the *Nae*I site is shown. **(B)** Percent deamination of each continuous strand C for all Exo1 generated gaps, as determined in panel A, reveals four regions resistant to deamination by the single-strand specific APOBEC3A cytidine deaminase. Percent accessible deamination is the average percentage deamination of all sites that are not present in the resistant regions. **(C)** Secondary structures of the continuous DNA strand for the four deamination resistant regions as predicted by UNAFOLD. The levels of cytidine deamination seen are displayed on the predicted secondary structure using colored boxes. **(D)** Relative deamination for all cytidines in all of the Exo1 excision tracts reveals a bias for deamination of cytidines in TC and CC dinucleotides over AC and GC dinucleotides. No similar bias is observed when considering cytidines and the following base.

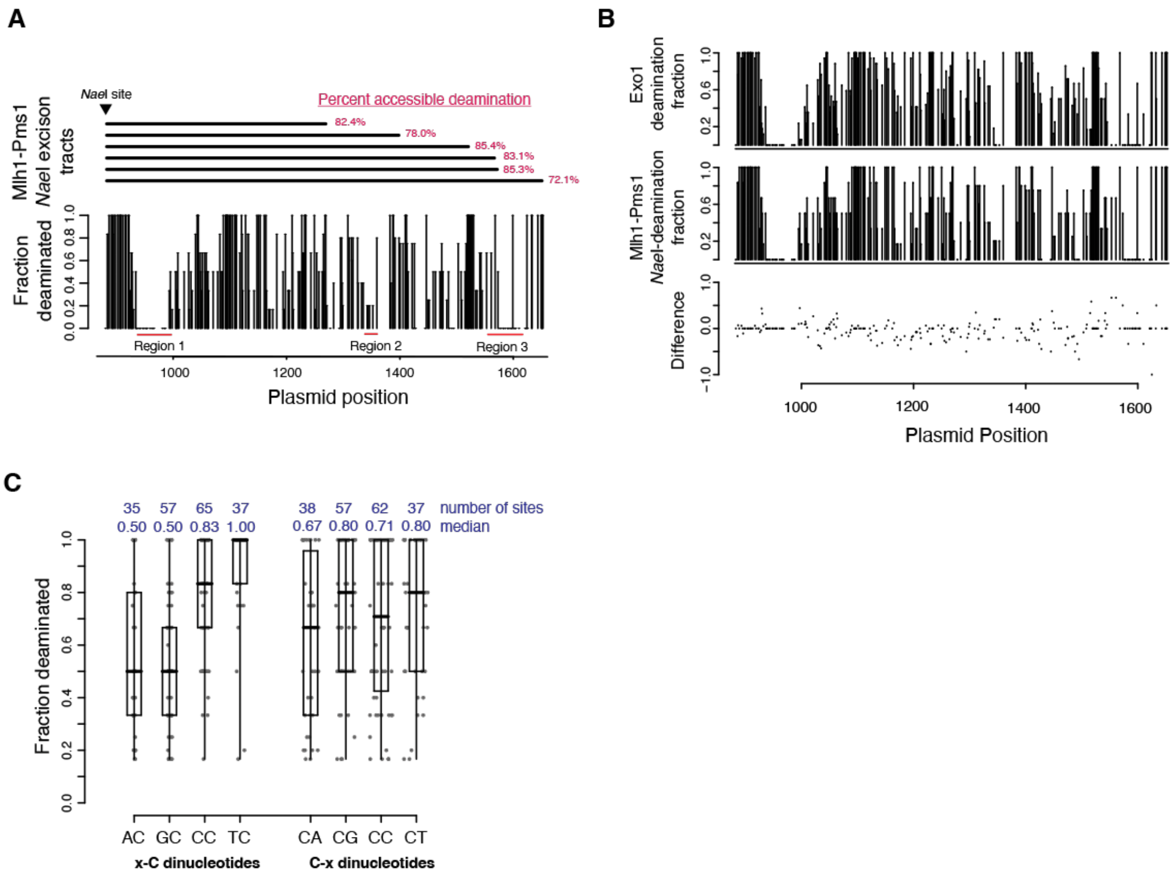

Supplemental Figure 5. APOBEC3A-mediated deamination tracts of products from the Mlh1-Pms1 excision reaction that start at the *Nae*I site. (A) Percent deamination of each continuous strand C for all plasmids recovered shares the regions resistant to deamination by the single-strand specific APOBEC3A cytidine deaminase in the Exo1 excision products (**Fig. S3B**). Percent accessible deamination is the average percentage deamination of all sites that are not present in the resistant regions. **(B)** Percent deamination for the products from Exo1 excision (top) are very similar to the products from Mlh1-Pms1 excision that start at the *Nae*I site (middle), and the differences between the two sets of samples tend to be at zero (bottom). **(C)** Relative deamination for all cytidines in the Mlh1-Pms1 excision tracts that start at the *Nae*I site reveals a bias for deamination of cytidines in TC and CC dinucleotides compared to AC and GC dinucleotides. No similar bias is observed when considering cytidines and the following base.

Supplemental Table 1. Oligonucleotides used for the construction of *Pst*I substrates.

Oligonucleotide	Sequence
1	ccgctctagaactagtggatccgcctcgactgcagattgggcccggtacccaattcgccc
2	gggcgaattgggtaccgggcccaatctgcagtcgaggcggatccactagttctagagcgg
3	gggcgaattgggtaccgggcccaatctgcaTgtcgaggcggatccactagttctagagcgg
4	gggcgaattgggtaccgggcccaatctgcaT
5	gtcgaggcggatccactagttctagagcgg
6	gggcgaattgggtaccgggcccaatctgca
7	Tgtcgaggcggatccactagttctagagcgg
8	gggcgaattgggtaccgggcccaatctgcaCtcgaggcggatccactagttctagagcgg
9	gggcgaattgggtaccgggcccaat
10	tcgaggcggatccactagttctagagcgg
11	gggcgaattgggtaccgggcccaatctgca
12	Ctcgaggcggatccactagttctagagcgg
13	gggcgaattgggtaccgggcccaatctgcaC
14	tcgaggcggatccactagttctagagcgg
15	gggcgaattgggtaccgggcccaatctgcaTg
16	tcgaggcggatccactagttctagagcgg
17	gggcgaattgggtaccgggcccaatctgcaTgt
18	cgaggcggatccactagttctagagcgg
19	gggcgaattgggtaccgggcccaatctgc
20	aTgtcgaggcggatccactagttctagagcgg
21	gggcgaattgggtaccgggcccaatctg
22	caTgtcgaggcggatccactagttctagagcgg
23	gggcgaattgggtaccgggcccaatctgcaTgtcga
24	ggcggatccactagttctagagcgg
25	gggcgaattgggtaccgggcccaat
26	ctgcaTgtcgaggcggatccactagttctagagcgg
27	gggcgaattgggtaccgggcccaatc
28	gattgggcccggtacccaattcgccc
29	ccgctctagaactagtggatccgcctcgactgca

Supplemental Table 2. Oligonucleotide combinations used to construct *Pst*I substrates.

**Substrate**	**Top strand oligonucleotides**	**Bottom strand oligonucleotides**
Homoduplex	2	1
Heteroduplex +T	3	1
Heteroduplex +T, 5nt 5′ nick	25, 26	1
Heteroduplex +T, 2nt 5′ nick	21, 22	1
Heteroduplex +T, 1nt 5′ nick	19, 20	1
Heteroduplex +T, 5′ nick	6, 7	1
Heteroduplex +T, 3′ nick	4, 5	1
Heteroduplex +T, 1nt 3′ nick	15, 16	1
Heteroduplex +T, 2nt 3′ nick	17, 18	1
Heteroduplex +T, 5nt 3′ nick	23, 24	1
Heteroduplex +T, bottom strand nick	3	28, 29
Heteroduplex CC	8	1
Heteroduplex CC, 5′ nick	11, 12	1
Heteroduplex CC, 3′ nick	13, 14	1
4nt gap	27, 5	1
5nt gap	9, 5	1
6nt gap	9, 10	1
